# Inhibitory effects of *Dendrobium candidum* Wall ex Lindl. on azoxymethane- and dextran sulfate sodium-induced colon carcinogenesis in C57BL/6 mice

**DOI:** 10.3892/ol.2013.1728

**Published:** 2013-12-04

**Authors:** QIANG WANG, PENG SUN, GUIJIE LI, KAI ZHU, CUN WANG, XIN ZHAO

**Affiliations:** Department of Biological and Chemical Engineering, Chongqing University of Education, Chongqing 400067, P.R. China

**Keywords:** *Dendrobium candidum* Wall ex Lindl., cytokine, C57BL/6 mice, colon carcinogenesis

## Abstract

*Dendrobium candidum* Wall ex Lindl. was purchased for the evaluation of azoxymethane (AOM)- and dextran sulfate sodium (DSS)-induced colon carcinogenesis in C57BL/6 mice. The body weights of the AOM- and DSS-induced colon cancer control groups were lighter than those of the untreated mice. *D. candidum* increased the body weights of the mice compared with the control group, and reduced the levels of the serum proinflammatory cytokines, IL-6, IL-12, TNF-α and IFN-γ, compared with the colon cancer control group. Reverse transcription-polymerase chain reaction and western blot analyses of the apoptotic-related genes, bax, bcl-2, caspase-3 and caspase-9, were performed in the colon tissues. The high-concentration *D. candidum* group showed a significant increase in the mRNA and protein expression levels of bax, caspase-3 and caspase-9 and decreased expression levels of bcl-2 compared with the control group. These results indicate that *D. candidum* Wall ex Lindl. exhibits preventive effects against colon carcinogenesis in mice.

## Introduction

*Dendrobium* is the second largest genus in the family Orchidaceae, exhibiting a vast diversity of floral and vegetative characteristics and being of considerable importance due to its broad geographic distribution and high-value hybrids bought as a floricultural commodity (1). The stems of the *Dendrobium* species are known as Shih-hu in Chinese, as sekkoku in Japanese and as *Dendrobium* stems in English (2). *Dendrobium candidum* Wall ex Lindl., a sympodial epiphytic orchid, is one of the most famous orchids with its distribution limited to a few countries in Southeast and South Asia (3). The dried stems of *D. candidum* are used in traditional Chinese or folk medicine as Yin tonic to strengthen stomach capacity or to promote body fluid secretion, prevent cataract development, relieve throat inflammation and fatigue, reduce peripheral vascular obstruction and enhance immunity (4). Previous pharmaceutical studies have been concentrated on the beneficial activities of *D. candidum,* including its antihyperthyroidism and anticancer effects (5). *D. candidum* contains water-soluble polysaccharides, phenanthrenes and a number of amino acids. The types of *Dendrobium* with a high chrysotoxum and erianin content may aid the inhibition of liver cancer and Ehrlich’s ascites carcinoma cell growth (4).

Colorectal cancer is a type of cancer that arises from uncontrolled cell growth in the colon, rectum or appendix. The symptoms of colorectal cancer typically include rectal bleeding and anemia, which are associated with changes in bowel habits and weight loss (6). The correlation between colitis and colorectal cancer is currently broadly accepted, and chronic inflammation is assumed to be a direct cause of colitis-associated cancer (7).

Apoptosis induction in cancer cells is initially identified by morphological changes, including cell shrinkage, membrane blebbing, chromatin condensation and nuclear fragmentation (8). Apoptosis is an important defense against cancer. Elucidating the critical events associated with carcinogenesis provides an opportunity for preventing cancer development by inducing apoptosis, particularly with bioactive agents or folk medicine. Chinese folk medicine is a significant environmental factor in the overall cancer process and exacerbates or interferes with disease progression (9).

The present study examined the preventive effect of *D. candidum* Wall ex Lindl on colon carcinogenesis. The inflammation-related cytokines, interleukin (IL)-6, IL-12, tumor necrosis factor (TNF)-α and interferon (IFN)-γ, were used to analyze these preventative effects on azoxymethane (AOM)- and dextran sulfate sodium (DSS)-induced colon carcinogenesis in mice. Gene expression was also used to determine these preventative effects *in vivo*.

## Materials and methods

### Preparations of D. candidum Wall ex Lindl

*D. candidum* was purchased at Shanghai Pharmacy Co., Ltd. (Shanghai, China). The *D. candidum* was stored at −80°C and freeze-dried to produce a powder. A 20-fold volume of methanol was added to the powdered sample and extracted twice by stirring overnight. The methanol extract was evaporated using a rotary evaporator (Eyela N-1100; Tokyo Rikakikai Co., Ltd., Tokyo, Japan), concentrated and then dissolved in dimethylsulfoxide (Amresco LLC, Solon, OH, USA) to adjust it to the required stock concentration (20%; w/v).

### Animals

Female C57BL/6 mice (n=40; 7 weeks old) were purchased from the Experimental Animal Center of Chongqing Medical University (Chongqing, China). The mice were maintained in a temperature controlled facility (temperature, 25±2°C; relative humidity, 50±5%) with a 12-h light/dark cycle and free access to a standard rat chow diet and water.

### AOM- and DSS-induced colon carcinogenesis model

The untreated group of mice received a common diet and water for the duration of the experimental period. The control group of mice were induced by AOM and DSS and were not treated with *D. candidum*. Solutions of *D. candidum* (200, 400 and 800 mg/kg) were administered to three sample groups, respectively, by gavage for the duration of the experimental period. Following *D. candidum* treatment for two weeks, the treatment and control groups were administered single intraperitoneal injections of AOM (10 mg/kg; Sigma-Aldrich, St. Louis, MO, USA). Between 2 and 5 weeks after the injections, the animals received 2.5% DSS (30,000–50,000 Mw; MP Biomedicals, LLC, Solon, OH, USA) in their drinking water for 7 days (10). The mice were then anesthetized with carbon dioxide and sacrificed. Blood and colon tissues were collected and preserved at −70°C until biological assays were performed. Mice were checked for body weight and colon length and weight. These experiments followed a protocol approved by the Animal Ethics Committee of Chongqing Medical University (Chongqing, China).

### Analysis of inflammation-related cytokines in serum by ELISA

For the serum cytokine assay, blood from the inferior vena cava was collected into a tube and centrifuged (730 × g; 10 min; 4°C). The serum was aspirated and assayed as described later. Serum concentrations of the inflammatory-related cytokines, IL-6, IL-12, TNF-α and IFN-γ (BioLegend, San Diego, CA, USA), were measured by ELISA according to the manufacturer’s instructions (BioLegend). Briefly, following the addition of biotinylated antibody reagent in 96-well plates, supernatants of homogenized serum were incubated at 37°C in CO_2_ for 2 h. Following washing with phosphate-buffered saline (PBS), streptavidin-horseradish peroxidase (HRP) solution was added and the plate was incubated for 30 min at room temperature. Absorbance was measured at 450 nm with an iMark microplate reader (Bio-Rad, Hercules, CA, USA) (11).

### Analysis of the serum levels of superoxide dismutase (SOD)

The total SOD assay kit (BioLegend) contained all reagents and solutions required for determining SOD activity in an indirect assay method based on xanthine oxidase and a novel color reagent. The chemical and biochemical properties of the color reagent used in the kit guaranteed a convenient application and linearity of test results compared among a broad range. For the blood biochemical assay, blood from the inferior vena cava was collected into a tube and centrifuged (730 × g; 10 min; 4°C). The serum level of SOD was determined using commercially available kits (Asan Pharm, Seoul, South Korea). Briefly, following the addition of biotin-antibody reagent in 96-well plates, supernatants of homogenized colon tissue were incubated at 37°C in CO_2_ for 1 h. Following the aspiration of each well and washing, HRP-avidin reagent was added to each well and incubated for 1 h at 37°C. The absorbance was measured at 450 nm using a microplate reader.

### Reverse transcription-polymerase chain reaction (RT-PCR) of apoptotic-related gene expression in the colon tissue

Total RNA was isolated from the colon tissue using TRIzol reagent (Invitrogen Life Technologies, Carlsbad, CA, USA), according to the manufacturer’s instructions. The RNA was digested with RNase-free DNase (Roche Diagnostics, Basel, Switzerland) for 15 min at 37°C and purified using a RNeasy kit (Qiagen, Hilden, Germany), according to the manufacturer’s instructions. cDNA was synthesized from 2 μg total RNA through incubation at 37°C for l h with avian myeloblastosis reverse transcriptase (GE Healthcare, Amersham, UK) and random hexanucleotides, according to the manufacturer’s instructions. The primers used to specifically amplify the genes of importance were for Bax (forward: 5′-AAG CTG AGC GAG TGT CTC CGG CG-3′, reverse: 5′-CAG ATG CCG GTT CAG GTA CTC AGT C-3′), Bcl-2 (forward: 5′-CTC GTC GCT ACC GTC GTG ACT TGG-3′, reverse: 5′-CAG ATG CCG GTT CAG GTA CTC AGT C-3′), caspase-3 (forward: 5′-CAA ACT TTT TCA GAG GGG ATC G-3′, reverse: 5′-GCA TAC TGT TTC AGC ATG GCA-3′) and caspase-9 (forward: 5′-GGC CCT TCC TCG CTT CAT CTC-3′, reverse: 5′-GGT CCT TGG GCC TTC CTG GTA T-3′). Equal amounts of RNA (1 μg) were reverse transcribed in a master mix containing 1X reverse transcriptase buffer, 1 mM dNTPs, 500 ng oligodT18 primers, 140 units MMLV reverse transcriptase and 40 units RNase inhibitor for 45 min at 42°C. PCR was then performed in an automatic thermocycler for 25 cycles (94°C for 30 sec, 55°C for 30 sec and 72°C for 40 sec) followed by an 8 min extension at 72°C. The amplified PCR products were run in 1.0% agarose gels and visualized by ethidium bromide staining (12).

### Protein extraction and western blot analysis in the colon tissue

Total cell lysates were obtained with an extraction buffer as previously described (13). Protein concentrations were determined using a protein assay kit (Bio-Rad, Hercules, CA, USA). For western blot analysis, the cell lysates were separated by 12% SDS-PAGE, transferred onto a polyvinylidene fluoride membrane (GE Healthcare), blocked with 5% skimmed milk and incubated with the primary antibodies (1:1,000 dilution). The mouse monoclonal antibodies against Bax, Bcl-2, caspase-3 and caspase-9 were obtained from Santa Cruz Biotechnology, Inc. (Santa Cruz, CA, USA). Following incubation with the horseradish peroxidase-conjugated secondary antibody at room temperature, immunoreactive proteins were detected using an enhanced chemiluminescence assay kit (GE Healthcare), according to the manufacturer’s instructions. Bands in the blot were visualized using a LAS3000 luminescent image analyzer (Fujifilm, Tokyo, Japan).

### Statistical analysis

Data are presented as the mean ± SD. Differences between the mean values for individual groups were assessed by one-way ANOVA with Duncan’s multiple range test. P<0.05 was considered to indicate a statistically significant difference. SAS version 9.1 (SAS Institute Inc., Cary, NC, USA) was used for the statistical analyses.

## Results

### Changes in body weight

**Figure 1:**
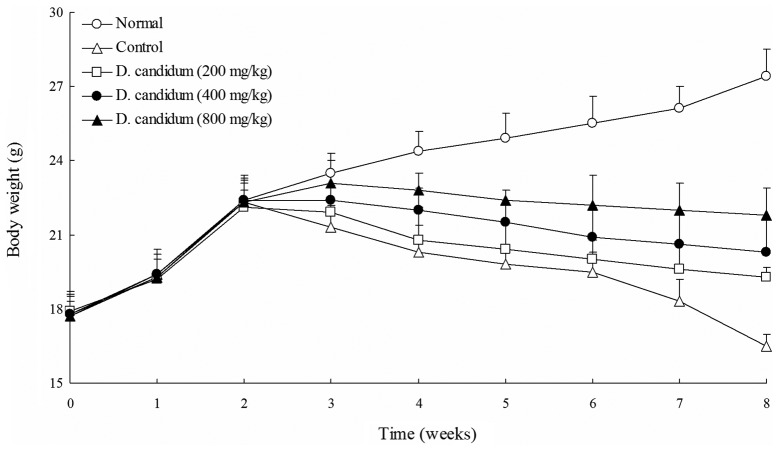
Effect of *Dendrobium candidum* Wall ex Lindl. on the weight changes in AOM- and DSS-induced colon carcinogenesis in C57BL/6 mice. AOM, azoxymethane; DSS, dextran sulfate sodium.

The untreated mice, in a normal dietary situation, did not exhibit reduced body weights. The body weights of the AOM- and DSS-induced colon carcinogenesis control mice were significantly decreased following the induction of colon carcinogenesis. As shown in Fig. 1, following the initiation of AOM- and DSS-induced colon carcinogenesis, the body weights of all mice in the AOM- and DSS-treated *D. candidum* groups were significantly lower than those of the mice in the untreated group. The 800 mg/kg *D. candidum* group of mice exhibited higher body weights than those of the 200 and 400 mg/kg *D. candidum* groups of mice.

### Changes in colon weight and length

The colon weight of the control group of mice was considerably higher than that of the untreated group of mice. The colon weights of the *D. candidum* groups were increased compared with the untreated group, but lighter than the control group (Table I). The high-concentration *D. candidum* group (800 mg/kg) of mice showed similar colon weights to the untreated group of mice. The total colonic length was significantly reduced in the AOM- and DSS-treated mice, as shown in Table I. The untreated group showed the longest colon length and the control group showed the shortest. The total colonic length was increased in the 800 mg/kg *D. candidum*-treated group compared with the 400 and 200 mg/kg *D. candidum*-treated groups. The colonic length was significantly shorter in AOM- and DSS-treated mice, which indicated that AOM and DSS contributed to the process of edematous changes in the colon in AOM and DSS colon carcinogenesis.

### Effect of D. candidum on the serum levels of IL-6, IL-12, TNF-α and IFN-γ

**Figure 2:**
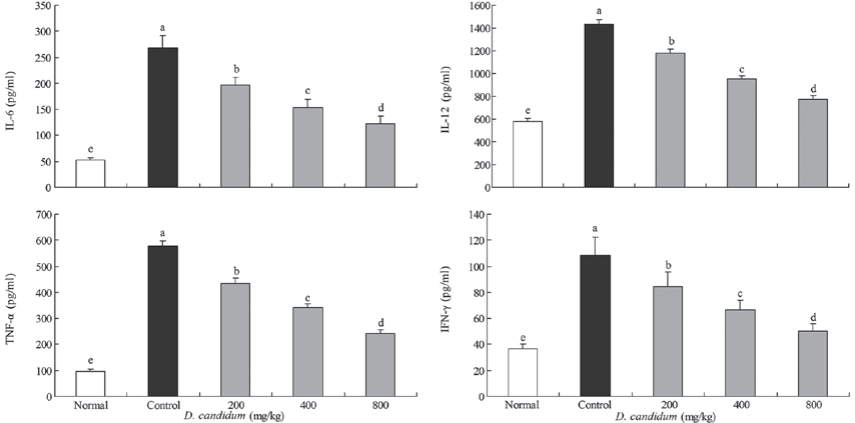
Serum IL-6, IL-12, TNF-α and IFN-γ levels of AOM- and DSS-induced colon carcinogenesis in mice treated with *Dendrobium candidum* Wall ex Lindl. ^a–e^Mean values with different letters over the bars are significantly different (P<0.05), according to Duncan’s multiple range test. AOM, azoxymethane; DSS, dextran sulfate sodium; IL, interleukin; TNF, tumor necrosis factor; IFN, interferon.

The IL-6 level in the untreated group of mice was 52.2±4.1 pg/ml. However, the IL-6 level in the control group of mice was significantly increased to 268.6±22.7 pg/ml. The levels of IL-6 in the mice treated with 200, 400 and 800 mg/kg *D. candidum* were 197.4±14.3, 152.6±16.4 and 122.4±14.4 pg/ml, respectively (Fig. 2). The IL-12 levels of the untreated, control and 200, 400 and 800 mg/kg *D. candidum-*treated mice were 578.6±27.7, 1,431.6±44.5 and 1,181.6±31.2, 954.4±26.8 and 774.5±31.3 pg/ml, respectively. The TNF-α levels in the untreated, control and 200, 400 and 800 mg/kg *D. candidum*-treated mice were 97.6±7.4, 577.2±21.2 and 435.6±19.7, 342.6±14.6 and 241.8±13.7 pg/ml, respectively. The IFN-γ levels in the untreated group of mice were the lowest at 36.9±3.6 pg/ml. The 200, 400 and 800 mg/kg *D. candidum-*treated mice showed higher levels of IFN-γ at 84.3±11.1, 66.3±7.5 and 50.3±5.5 pg/ml, respectively, compared with the untreated group of mice. The control group of mice showed the highest levels of IFN-γ at 108.4±14.2 pg/ml. The serum IL-6, IL-12, TNF-α and IFN-γ levels in the mice of the *D. candidum*-treated groups were significantly lower than those of the control group.

### Effect of D. candidum on the serum levels of SOD

**Figure 3:**
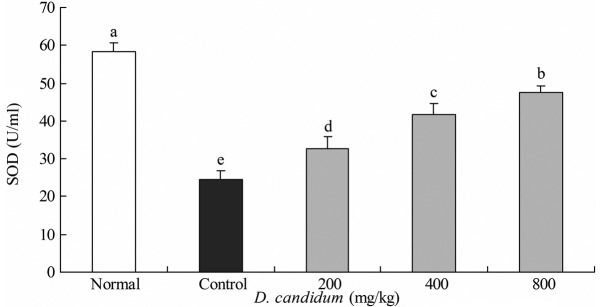
Serum superoxide dismutase (SOD) levels of AOM- and DSS-induced colon carcinogenesis in *D. candidum* Wall ex Lindl-treated mice. ^a–e^Mean values with different letters over the bars are significantly different (P<0.05), according to Duncan’s multiple range test. AOM, azoxymethane; DSS, dextran sulfate sodium.

The SOD level in the untreated group of mice was 53.8±2.4 U/ml. The control group of mice showed the lowest level of SOD at 24.6±2.2 U/ml, while the 200, 400 and 800 mg/kg *D. candidum*-treated mice exhibited increased levels of SOD at 32.7±3.2, 41.8±2.7 and 47.6±1.8 U/ml, respectively (Fig. 3).

### Apoptotic-related gene expression of Bax, Bcl-2 and caspases

**Figure 4:**
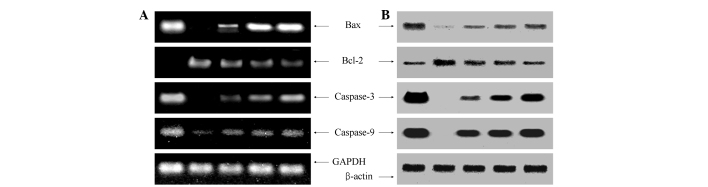
Effects of *Dendrobium candidum* Wall ex Lindl. on (A) mRNA and (B) protein expression levels of Bax, Bcl-2 and caspase-3 and-9 in the mouse colon tissues.

To elucidate the mechanisms underlying cancer prevention, the expression of Bax, Bcl-2, caspase-3 and caspase-9 in the colon tissues was measured by RT-PCR and western blot analyses. As shown in Fig. 4, the expression of proapoptotic Bax and antiapoptotic Bcl-2 showed significant changes in the presence of 800 mg/kg *D. candidum*. These results indicated that *D. candidum* induced apoptosis in the colon tissues of the AOM- and DSS-induced colon carcinogenesis mouse model via a Bax- and Bcl-2-dependent pathway. The mRNA and protein expression levels of caspase-3 and caspase-9 were extremely low in the control mouse tissues, but significantly increased following treatment with 800 mg/kg *D. candidum*. With the *D. candidum* treatment, the mRNA and protein expression of caspase-9 and caspase-3 was gradually elevated with increasing concentrations. Specifically, apoptosis induction by *D. candidum* was associated with the upregulation of Bax, caspase-3 and caspase-9 and the downregulation of Bcl-2 in terms of mRNA and protein expression. The anticancer effect of 800 mg/kg *D. candidum* treatment was greater than that of the 200 and 400 mg/kg *D. candidum* treatments.

## Discussion

Although *D. candidum* has been previously used as a medicine, little scientific data on its effects are available. *D. candidum* has been previously reported to exhibit various therapeutic effects on numerous pathological conditions, including inflammation, immunity and cancer (14).

The most important symptoms of the AOM- and DSS-induced colon carcinogenesis in mice are body weight loss, colon length shortening and colon weight increase (10). Colon length may be measured to determine the severity of colon carcinogenesis. Changes in colon weight and length reflect the carcinogenesis status of mice with AOM- and DSS-induced colon carcinogenesis, and also demonstrate which concentration has a better preventive effect on AOM- and DSS-induced colon carcinogenesis (15).

Tumor-associated inflammatory cytokines, such as IL-6 and TNF-α, are likely to regulate cancer cells in the tumor microenvironment (16). Previous studies investigating the benefit of IL-12 to antitumor immunity provide further insight into the physiologically relevant stimuli for IFN-γ production to enhance anticancer immunity (17). IFN-γ has a profound impact on solid tumor growth and metastasis and appears to play an early role in the protection from metastasis (18). Lower levels of IL-6, IL-12, TNF-α and IFN-γ are indicative of improved anticancer effects (10). SOD is an important antioxidative enzyme that catalyzes the dismutation of the superoxide anion into hydrogen peroxide and molecular oxygen (19). One benefit of SOD is cancer prevention, and another is that is useful for preventing the damage and side-effects that arise from cancer therapies, such as radiotherapy and chemotherapy (20).

Apoptosis is a fundamental cellular event, and understanding its mechanisms of action will aid the harnessing of this process for use in tumor diagnosis and therapy (21). The antiapoptotic gene, Bcl-2, is expressed on the outer mitochondrial membrane surface (22). Since the Bax and Bcl-2 genes are mainly expressed during apoptosis, we hypothesize that these genes regulate apoptotic activity. Apoptosis results from the activation of caspase family members that act as aspartate-specific proteases (23). Caspases form a proteolytic network within the cell, whereby upstream initiator caspases are activated early in the apoptotic process (caspase-9) and in turn, activate other downstream caspases (caspase-3). Cytochrome-*c* and procaspase-9 processing is highly dependent on caspase-3, allocating this caspase in a central position as a regulator of essential apoptotic pathways in cancer cells (24).

The present study demonstrated that *D. candidum* is effective in the prevention of AOM and DSS-induced colon cancer in mice. The results show that the anticancer effects of *D. candidum* increased the serum SOD level and decreased the levels of pro-inflammatory cytokines IL-6, IL-12, TNF-α and IFN-γ. Furthermore, mRNA and protein expression levels of apoptotic genes in the colon tissues, including Bax, Bcl-2, caspase-3 and caspase-9 were determined. These results suggest that *D. candidum* is potentially useful in the prevention of chemical-induced colon cancer.

## Figures and Tables

**Table I:** Effect of *Dendrobium candidum* Wall ex Lindl. on the changes in colon weight and length in AOM- and DSS-induced colon carcinogenesis in C57BL/6 mice.

Group	Colon weight, g	Colon length, mm
Untreated	0.29±0.04^d^	80.37±3.54^a^
Control	0.45±0.05^a^	68.25±3.47^d^
*D. candidum* Wall ex Lindl., mg/kg		
200	0.42±0.03a,b	71.32±3.21c,d
400	0.39±0.04^b^	73.04±4.03^c^
800	0.34±0.03^c^	77.28±4.22^b^

a–d Mean values with different letters in the same column are significantly different (P<0.05), according to Duncan’s multiple range test. Data are presented as the mean ± SD.

AOM, azoxymethane; DSS, dextran sulfate sodium.
